# Possibility of Recycling SiO_x_ Particles Collected at Silicon Ingot Production Process as an Anode Material for Lithium Ion Batteries

**DOI:** 10.1038/s41598-019-50011-8

**Published:** 2019-09-16

**Authors:** Junghyun Kim, So Yeun Kim, Cheol-Min Yang, Gyo Woo Lee

**Affiliations:** 1 Division of Mechanical Design Engineering, Chonbuk National University, Jeonju, 54896 Republic of Korea; 2 Institute of Advanced Composite Materials, Korea Institute of Science and Technology (KIST), Wanju, Jeollabuk-do 55324 Republic of Korea

**Keywords:** Electronic devices, Batteries

## Abstract

Recently, some studies have utilized silicon (Si) as an anode material of lithium ion battery by recycling Si from the slurry of wafer slicing dust. The filtration of Si particles condensed from Si vapors that were exhausted from the ingot growing furnace could propose another method of Si recycling. In this study, we investigated the possibility of using such collected silicon oxides (SiO_x_) particles as an anode material. After collecting SiO_x_ particles, FE-SEM, TEM, EDS, XRD, XPS analysis, and charge/discharge test were carried out to investigate characteristics and usability of these particles. FE-SEM and FE-TEM images showed that these particles mainly consisted of spherical primary particles with a diameter of 10 nm or less. Agglomerates of these primary particles were larger than 300 nm in diameter. In TEM image and EDS analysis, crystalline particles were observed along with amorphous particles. As a result of XRD analysis, amorphous silica (SiO_2_) and crystalline Si were observed. Charge/discharge tests were carried out to determine the feasibility of using these particles as an anode material for lithium ion batteries. A cycle efficiency of 40.6% was obtained in the test in which the total number of charge/discharge cycle was 100 under the condition of C-rate 0.2 for the first three times and C-rate 1.0 for the remaining 97 times. Results showed that these collected particles could be used as an anode material for lithium ion batteries.

## Introduction

Silicon (Si) has been widely used to fabricate semiconductors for electronic devices. It is utilized in the shape of a wafer made by melting raw polysilicon, growing in the form of a single crystal ingot, and then cutting the ingot thinly according to the purpose of use. The importance of renewable energy has been emphasized to reduce the consumption of fossil fuel. Si has also been used as a raw material for photovoltaic solar cells to utilize solar energy. With increasing need for solar cells due to the growth of photovoltaic cell market, demand for Si to produce wafer is steadily increasing^1,2^.

However, a large amount of Si loss occurs in the slicing process using wire saws when a thin wafer is cut out from the cylindrical Si ingot. This kerf loss might increase the production cost of Si wafers. The resulting Si sludge adversely affects the environment, resulting in additional costs for the sludge treatment. In general, Si sludge from single-crystal ingot is consisted of kerf-loss crystalline Si and some impurities like lubricant oil and metal debris from the wire saw. When the sludge is recycled the impurities need to be removed through complex and expensive procedures^3,4^. To reduce such loss of Si, several studies have tried to recycle Si from the sludge^3–9^. Wang *et al*.^5^ have recycled Si sludge to fabricate solar cells, and analyzed the performance of these cells. They reported that solar cells made from recycled Si could achieve a performance less than 2% compared to commercial solar cells, making it possible to use the recycled Si. Tsai^6,7^ has separated Si from impurities using electric force and particle weight. Tomono *et al*.^8^ have also studied the recycling of the produced sludge from a slicing process using diamond wires. Removal efficiencies of lubricant and metal impurities using acetone and acid solutions, respectively, were examined by thermal and elemental analyses. Sergiienko *et al*.^9^ have reported that Si sludge can be separated into Si and silicon carbide (SiC) by centrifugation and cyclone.

Various methods of secondary batteries have been explored in many researches throughout the years. Xie *et al*.^10^ reviewed the recent progress of Li-ion storage performances of various rhenium disulfide (ReS_2_) electrodes, mainly focusing on the synthesis method, structures, the reaction mechanism, and the corresponding electrochemical performance. In 2018, Liao *et al*.^11^ reported that the morphology, size and phase of the material (nano-sized niobium pentoxide) play a crucial role in its electrochemical performance, and discussed that the material has the great potential as a practical high-rate anode material for lithium-ion batteries. In the recent review paper, Wang *et al*.^12^ summarized the synthesis strategies, common characterization techniques, and their important role in electrochemical lithium/sodium ions storage and capacitance properties of oxygen-defective metal oxides.

In this study, we investigated the possibility of recycling the silicon oxides (SiO_x_) particles mixed with crystalline Si collected from Si vapors in the ingot growing process. It is expected that the coexistence of Si crystal and amorphous silica (SiO_2_) particles could be helpful to achieve good electrochemical performances as an anode material for lithium ion batteries. Additionally, the proposed approach is based on a facile, green, and cost-effective process.

## Results and Discussion

### Particle characteristics

**Figure 1:**
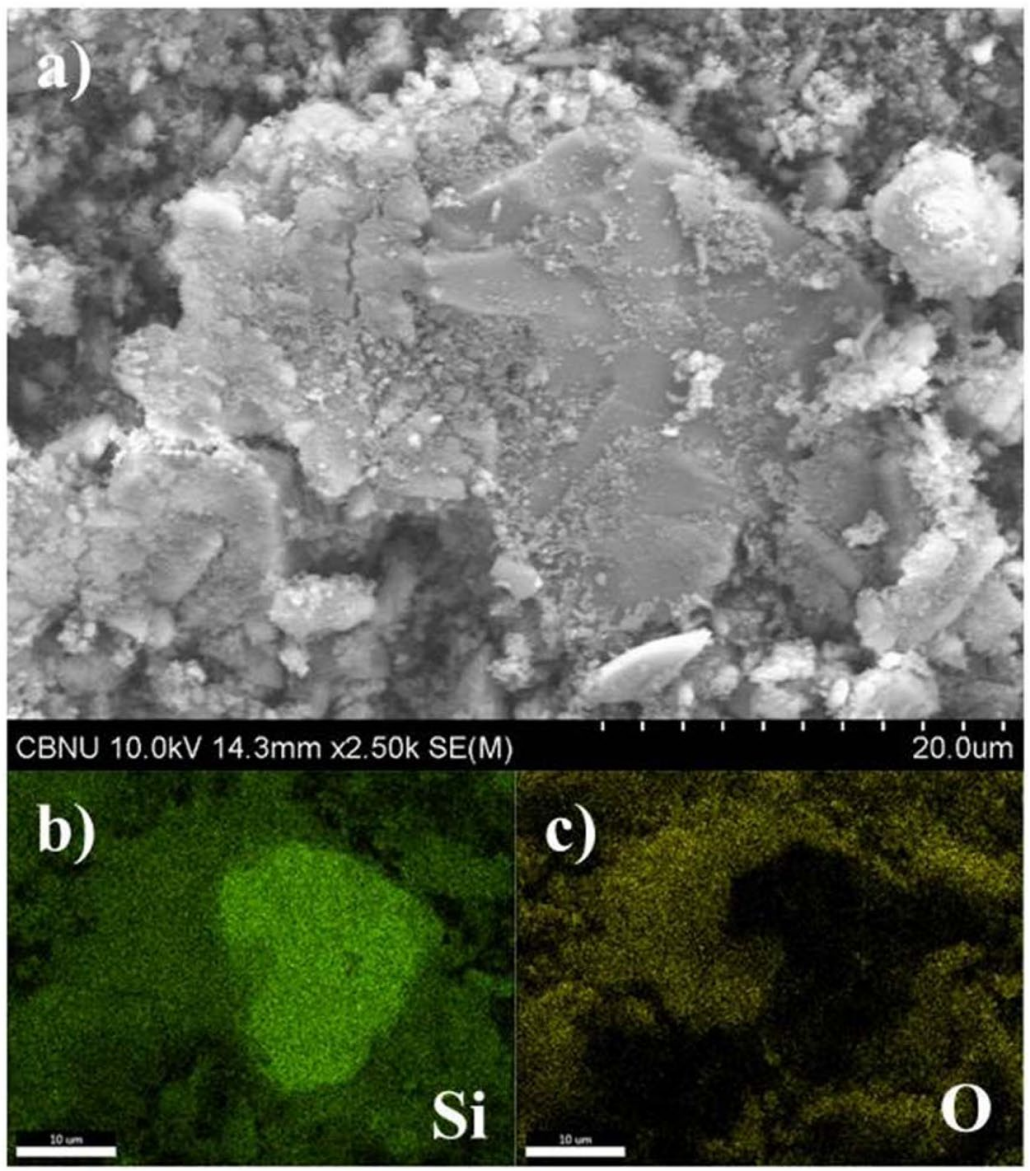
(**a**) FE-SEM image of the collected SiO_x_ particles, (**b**,**c**) EDS mappings of the collected SiO_x_ particles.

FE-SEM and EDS mapping images of the particles are shown in Fig. 1, including FE-SEM image of particles (Fig. 1a), Si element mapping (Fig. 1b), and O element mapping picture (Fig. 1c). Figure 1a has a scale bar of 20 μm and a particle size at the center is larger than 30 μm. Also, lots of nanometer scaled particles are seen around the large particles. FE-SEM image shows that these particles are a mixture of μm size particles and agglomerates of nm particles. EDS mapping photos in Fig. 1b and Fig. 1c show that regions of Si and O can be clearly distinguished. These mapping photos suggest that the particle larger than 30 μm in size in Fig. 1a might be Si crystal rather than SiO_x_.

The averages and standard deviations of weight and atomic fractions for Si and O elements of the samples from the EDS analyses are as follows: 53.8 wt% (std. dev. = 8.5 wt%) and 40.3 at% (std. dev. = 8.7 at%) for Si and 46.2 wt% (std. dev. = 8.5 wt%) and 59.7 at% (std. dev. = 8.7 at%) for O element, respectively.

**Figure 2:**
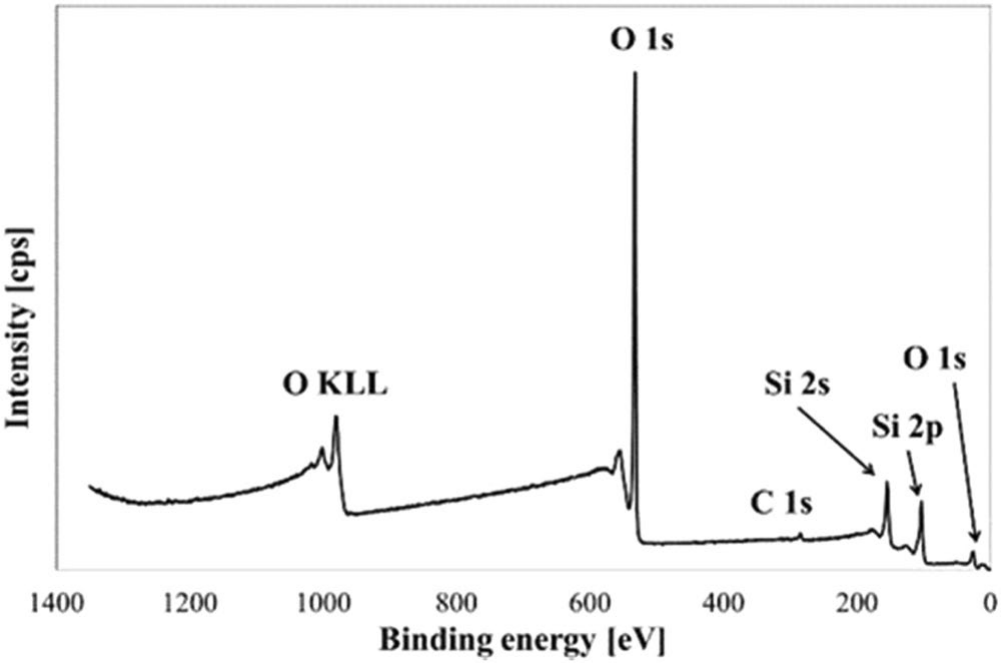
XPS spectra of SiO_x_ particles showing O, Si, and C elements.

To investigate the composition of particles, XPS spectrum was obtained as shown in Fig. 2. In the XPS spectrum, Si and O elements were identified as in EDS analysis. In addition to these two elements, a small amount of C element was detected. It was originated from the graphitic heater in the ingot grower. Based on the results of EDS and XPS analyses we believed that the collected particles are mainly consisted of silicon oxides (SiO_x_) and crystalline silicon (Si).

**Figure 3:**
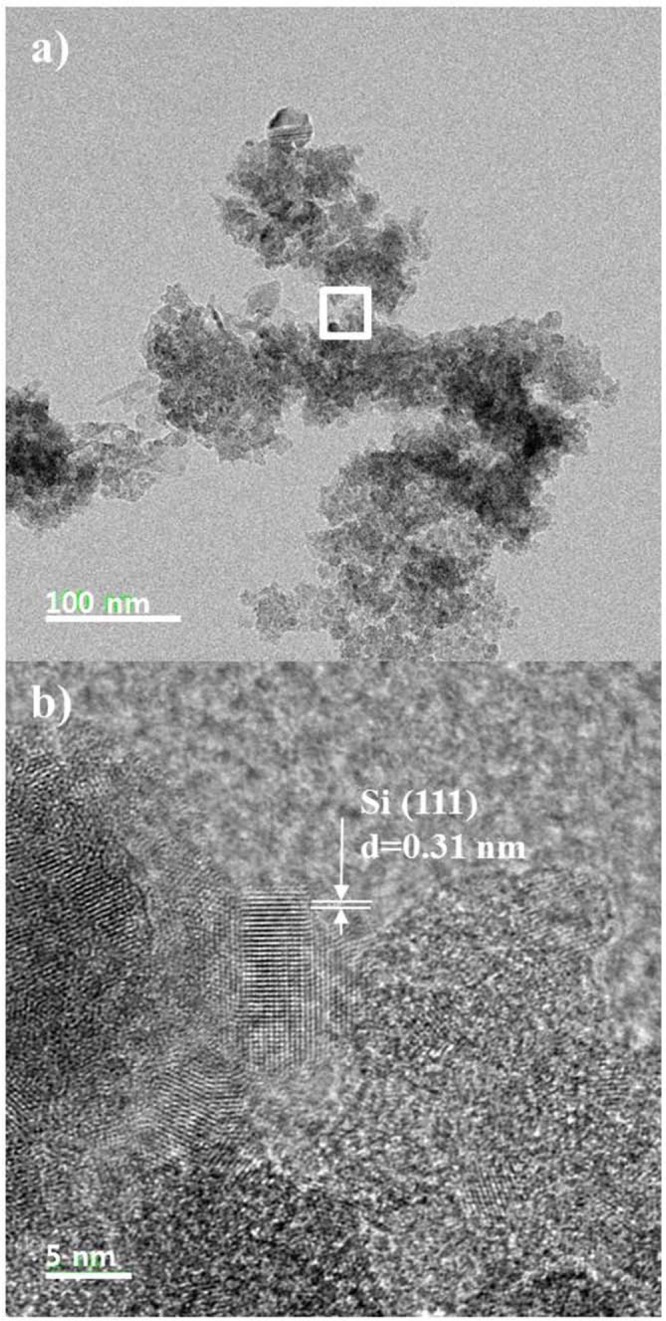
TEM images showing aggregated nanometer sized particles (**a**) and crystalline structures of particles with high magnification (**b**).

Figure 3 is TEM images of collected Si particles with crystalline structures. The scale bar is 100 nm in Fig. 3a and 5 nm in Fig. 3b. Agglomerates or aggregates of nanometer particles were approximately 300 nm in diameter. Figure 3b is an enlarged view of a rectangular portion seen at the central position of Fig. 3a. In the figure, the size of primary particles was less than 10 nm, and the lattice spacing was 0.31 nm. Crystalline particles could be found along with amorphous SiO_x_ particles.

**Figure 4:**
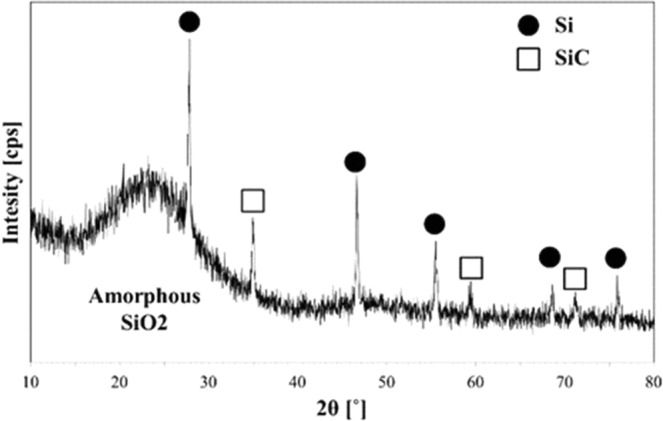
XRD peaks showing Si and SiC crystalline and amorphous SiO_2_.

Figure 4 is the XRD spectrum showing peaks of Si crystals, amorphous SiO_2_ particles, and SiC. The broad peak at 2θ equals 20° ~ 30° on the left side of Fig. 4, showing the presence of amorphous SiO_2_. In case of larger than 30° for 2θ value several peaks confirm the presence of crystalline Si and SiC^13–16^. When the SiO_x_ particles were used as an anode material, it was known through the result of the previous study that the higher the Si crystal content, the higher the charge/discharge capacity^17^. In their article, Takezawa *et al*.^17^ showed that the values of charge and discharge capacities were decreased with increasing values of *x* in SiO_x_. On the basis of this literature and the XRD analysis we believe that it is quite possible to use the collected SiO_x_ powder as an anode materials of the lithium ion battery

**Figure 5:**
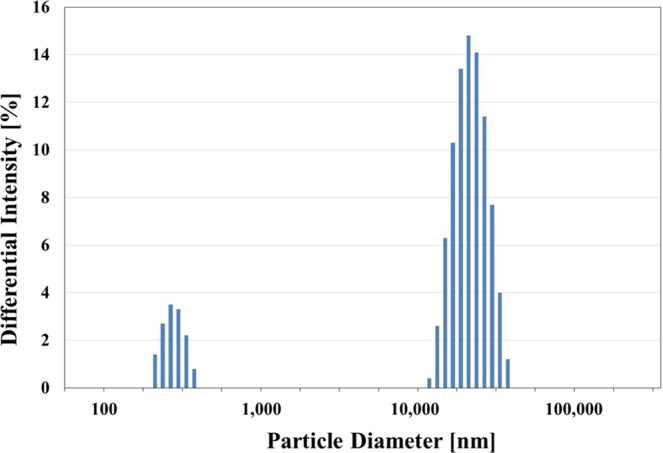
Particle size distribution showing a bimodal shape with mode values of 322.9 nm and 17.9 μm.

Figure 5 shows particle size distribution of collected particles. The distribution is bimodal with a group of nm sized particles and a group of μm sized particles. This could explain the μm sized particles shown in FE-SEM photographs and lots of agglomerated nm sized particles found around the μm sized particles. The mean sizes of the nm size group on the left and the μm size one on the right were 322.9 nm and 17.9 μm, with standard deviations of 40.7 nm and 3.5 μm, respectively. The specific surface area and average pore size of the particles measured by BET and BJH analyses were 184.4 m^2^/g and 7.4 nm, respectively. FE-SEM image, the specific surface area, and the pore size suggested that tens of nanometer particles aggregated to form larger particles with diameter of approximately 300 nm and agglomerates of larger particles formed tens of micrometer sized particles.

These collected particles were identified as SiO_x_ particles mixed with crystalline Si through several analyses. When Si is used as an anode material for a lithium ion battery, crystalline Si is advantageous in terms of the capacity of the battery. There have been some attempts to utilize amorphous SiO_x_ particles because of disadvantages caused by the expansion and contraction of crystalline Si particles occurred during charging and discharging process^18^. In this view, proper mixing of these two types of particles (Si crystal and amorphous SiO_2_) could be helpful to achieve good electrochemical performance when they are used as an anode material for lithium ion batteries.

### Electrochemical property

**Figure 6:**
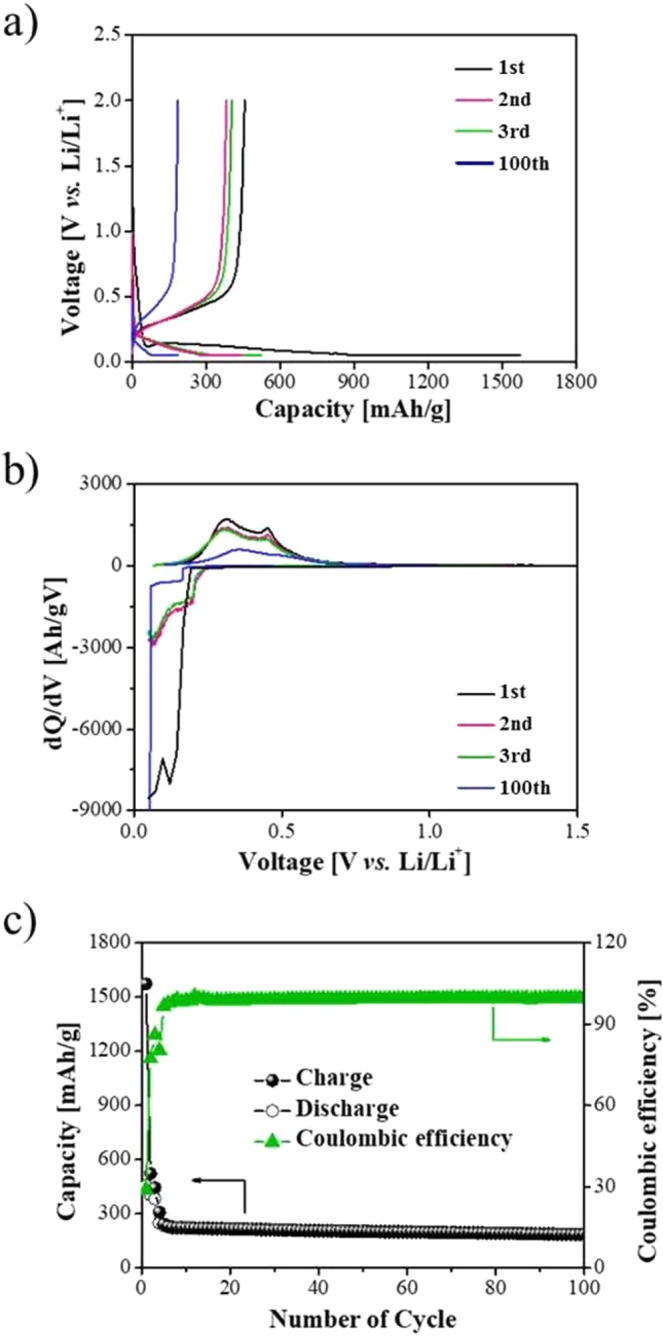
Electrochemical performance of SiO_x_ anode. (**a**) Galvanostatic charge and discharge profiles in the 1st, 2nd, 3rd, and 100th cycles. (**b**) Differential capacity vs. cell potential curves. (**c**) Cycle performance and coulombic efficiency of the half coin cell from 1 to 100 charge/discharge cycles.

Figure 6a shows the galvanostatic charge (Li^+^ insertion) and discharge (Li^+^ extraction) profiles of SiO_x_ anodes in the voltage range of 0.05 to 2.0 V (vs. Li/Li^+^) at room temperature. As a result of the charge/discharge test, the initial charging capacity was 1572.8 mAh/g and the initial discharging capacity was 456.3 mAh/g.

According to the differential capacity plots for the SiO_x_ electrodes Fig. 6b, a broad peak is visible at ~0.8 V, which is mainly associated with the SEI layer formation on the surface of the SiO_x_ particles during the first charge. When the potential reaches ~0.25 V vs Li/Li^+^, Si begins to react with Li^+^, resulting two delithiation peaks at ~0.3 and 0.45 V vs Li/Li^+^, which are characteristics for the lithiation reactions of amorphous Li_x_Si.

Figure 6c is a graph showing results of charge/discharge tests up to 100 cycles of the fabricated coin cell. The upper line of the graph shows coulombic efficiency change which means the ratio of the discharge capacity to the charge capacity. Lines at the bottom of the graph show change of charge and discharge capacity according to charge/discharge cycle. The battery capacity maintenance efficiency up to 100 cycles was 40.6% of the initial discharge capacity. This was lower than the average battery capacity retention efficiency (about 70%) of coin cells using modified Si as an anode material^13–16^. The coulombic efficiency was 29.0% at the first charge/discharge and close to 100% after the 5th cycle. It is necessary to increase the initial coulombic efficiency through modification of particles.

The reason why the initial coulombic efficiency was low during charging and discharging was due to reaction of Si and electrolyte. In this reaction, a layer called SEI was formed on the Si surface. During the reaction, Li^+^ was lost and the capacity was reduced. When SEI was formed, the reaction was terminated and Li^+^ was used in the charge and discharge processes to maintain a coulombic efficiency of about 99%. However, if SEI cracks, a new SEI is created, resulting in lower efficiency^18–20^. Based on charge/discharge test results, we believe that, if accompanied with surface modification, SiO_x_ particles collected from Si vapors in the ingot growing process could be utilized as an anode material of lithium ion battery.

## Summary and Conclusion

The purpose of this study was to investigate the possibility of using recycled SiO_x_ particles as an anode material of a lithium ion battery. FE-SEM, EDS, TEM, XRD, PSA, BET and XPS analyses were done on SiO_x_ particles collected at the vapor exhaust duct of the ingot growing furnace. In addition, a half coin cell was prepared using these collected particles as an anode material and electrical characteristics of the cell were measured through charge/discharge test. Through these analyses, it was confirmed that the collected particles were SiO_x_ particles mixed with crystalline Si and amorphous SiO_2_. These particles were distributed in two groups with average sizes of 322.9 nm and 17.9 μm. The specific surface area was 184.4 m^2^/g and the average pore size was 7.4 nm. In the charge/discharge test, the battery capacity maintenance efficiency was 40.6% compared to the initial discharge amount after up to 100 cycles and the coulombic efficiency was more than 99% after 5 cycles.

This study showed that the recycling SiO_x_ particles exhausted from the Si ingot grower is environmentally friendly and economical. Results also showed that these collected particles could be used as an anode material for lithium ion batteries.

## Experimental Details

### Collection of Si particles

Si particles were collected at a single crystal Si ingot grower made by S-Tech Co. Ltd. (Model SS1). The grower is currently operated by M company, one of domestic Si ingot producers in South Korea. The filtration system used for collecting Si particles was manufactured by H & D Co. Ltd. It is cylindrical in shape with an outer diameter of 558 mm, a length of 1315 mm, and a thickness of 8.5 mm. The filter installed for collecting particles was made by 3GS Co. Ltd. It has 10 mm thick glass fiber material and pores of 30 to 40 μm in diameter. The filter has 50 wrinkles with an inner diameter of 224 mm, an outer diameter of 359 mm, and a length of 535 mm. Details of particle collection were described in a previous work^21^.

### Characterization of collected particles

To determine particle shape and compositions, field-emission scanning electron microscopy (FE-SEM) images were obtained and energy dispersive spectrometer (EDS) analysis was performed, respectively (SU-70, HITACHI). Brunauer-Emmett-Teller (BET) and Barrett-Joyner-Halenda (BJH) analyses from N_2_ adsorption isotherms (77 K) measured using volumetric equipment were performed to confirm the specific surface area and pore size distribution of particles, respectively (ASAP2010, Micromeritics). Particle size analysis (PSA) was also performed to know the particle size distribution of particles (UPA-150, Microtrac). Cs-corrected field emission transmission electron microscopy (FE-TEM) analysis was also performed to confirm the crystal structure of particles (JEM-ARM-200F, JEOL). X-ray diffraction (XRD, MAX-2500, RIGAKU) and X-ray photoelectron spectroscopy (XPS, K-Alpha XPS System, Thermo Fisher Scientific) were used to observe crystalline structures.

### Measurement of electrochemical property

A half-coin cell (CR 2032) was fabricated to check the possibility of using these collected Si particles as an anode material of the lithium ion secondary battery. Electrode was prepared by mixing three components [active material: binder (35 wt% PAA, Aldrich): conductive agent (Super P, Fisher Scientific) = 8: 1: 1 by weight] to make a slurry which was then applied to copper foil and dried at 80 °C for 6 hours. The painted copper foil was squeezed using a roll press and dried under vacuum at 120 °C for 12 hours. After that, circular electrode plates (15 mm in diameter, according to coin cell size) were made from the painted and completely dried copper foil using a punching machine. The electrolyte solution was 1 M LiPF_6_ (Aldrich) in which ethylene carbonate (EC) and diethyl carbonate (DEC) were mixed at the same volume. WBCS3000Le32 tester (WonA Tech Co.) was used for charge/discharge test of the fabricated battery. Test conditions were set to 0.2 C rate for forming solid electrolyte interphase (SEI) up to the initial 3 cycles. Then 1.0 C rate was maintained for the following 97 cycles. The cut off condition was 0.05 to 2 V. It was the same for all 100 cycles.
